# Synergistic effects of NOD1 or NOD2 and TLR4 activation on mouse sickness behavior in relation to immune and brain activity markers^☆^

**DOI:** 10.1016/j.bbi.2014.08.011

**Published:** 2015-02

**Authors:** Aitak Farzi, Florian Reichmann, Andreas Meinitzer, Raphaela Mayerhofer, Piyush Jain, Ahmed M. Hassan, Esther E. Fröhlich, Karin Wagner, Evelin Painsipp, Beate Rinner, Peter Holzer

**Affiliations:** a Research Unit of Translational Neurogastroenterology, Institute of Experimental and Clinical Pharmacology, Medical University of Graz, Universitätsplatz 4, 8010 Graz, Austria; b Clinical Institute of Medical and Chemical Laboratory Diagnostics, Medical University of Graz, Auenbruggerplatz 15, 8036 Graz, Austria; c Core Facility Molecular Biology, Center for Medical Research, Medical University of Graz, Stiftingtalstrasse 24/1, 8010 Graz, Austria; d Core Facility Flow Cytometry, Center for Medical Research, Medical University of Graz, Stiftingtalstrasse 24/1, 8010 Graz, Austria

**Keywords:** Anxiety, c-Fos, Corticosterone, FK565, Food intake, Kynurenine, Lipopolysaccharide, Locomotion, Muramyl dipeptide, Proinflammatory cytokines

## Abstract

Toll-like receptors (TLRs) and nuclear-binding domain (NOD)-like receptors (NLRs) are sensors of bacterial cell wall components to trigger an immune response. The TLR4 agonist lipopolysaccharide (LPS) is a strong immune activator leading to sickness and depressed mood. NOD agonists are less active but can prime immune cells to augment LPS-induced cytokine production. Since the impact of NOD and TLR co-activation *in vivo* has been little studied, the effects of the NOD1 agonist FK565 and the NOD2 agonist muramyl dipeptide (MDP), alone and in combination with LPS, on immune activation, brain function and sickness behavior were investigated in male C57BL/6N mice.

Intraperitoneal injection of FK565 (0.001 or 0.003 mg/kg) or MDP (1 or 3 mg/kg) 4 h before LPS (0.1 or 0.83 mg/kg) significantly aggravated and prolonged the LPS-evoked sickness behavior as deduced from a decrease in locomotion, exploration, food intake and temperature. When given alone, FK565 and MDP had only minor effects. The exacerbation of sickness behavior induced by FK565 or MDP in combination with LPS was paralleled by enhanced plasma protein and cerebral mRNA levels of proinflammatory cytokines (IFN-γ, IL-1β, IL-6, TNF-α) as well as enhanced plasma levels of kynurenine. Immunohistochemical visualization of c-Fos in the brain revealed that NOD2 synergism with TLR4 resulted in increased activation of cerebral nuclei relevant to sickness.

These data show that NOD1 or NOD2 synergizes with TLR4 in exacerbating the immune, sickness and brain responses to peripheral immune stimulation.

Our findings demonstrate that the known interactions of NLRs and TLRs at the immune cell level extend to interactions affecting brain function and behavior.

## 1 Introduction

Activation of the immune system by microbial invasion results in the production of proinflammatory cytokines leading to sickness behavior, an adaptive behavioral response whose prototypical symptoms comprise a reduction of locomotor activity, anorexia, and anhedonia (Dantzer, 2004; Dantzer et al., 2008; Hart, 1988). Microbial invasion is sensed by cells of the innate immune system through activation of pattern-recognition receptors (PRRs), following the recognition of molecular structures specific for pathogens, termed pathogen-associated molecular patterns (PAMPs) (Kawai and Akira, 2011). The first identified and best characterized PRRs belong to the family of Toll-like receptors (TLRs); however, other PRRs such as the nuclear-binding domain (NOD)-like receptors (NLRs) represent a further group of PRRs playing important roles in PAMP recognition and immunity (Franchi et al., 2009; Kawai and Akira, 2011). Unlike NLRs which are intracellular PRRs, TLRs are associated with the cell membrane.

Lipopolysaccharide (LPS) is a major component of the outer membrane of Gram-negative bacteria and acts as a predominant TLR4 agonist (Poltorak et al., 1998; Kawai and Akira, 2011). After binding to TLR4 it leads to NF-κB and mitogen-activated protein (MAP) kinase activation and induces a strong cytokine response (Poltorak et al., 1998; Kawai and Akira, 2011). Thus, LPS is one of the most widely studied PAMPs triggering acute sickness behavior, as well as delayed depression-like behavior in rodents (Frenois et al., 2007; Yirmiya, 1996) and elicits similar effects to that of the injection of specific cytokines such as IL-1β (Anisman et al., 2008) and TNF-α (Bluthe et al., 1991).

The behavioral effects of peripheral immune activation are mediated via an afferent neural and an endocrine pathway. As part of the endocrine pathway, cytokines and circulating PAMPs reach the brain at the level of the choroid plexus and the circumventricular organs and induce the expression of cytokines within the brain (Dantzer et al., 2000). The peripheral and central effects of immune activation can be assessed by means of several parameters. First, immune activation induces c-Fos-like immunoreactivity, an indicator of neuronal activation, within the brain and can provide insights into the neural networks that subserve sickness symptoms (Gaykema and Goehler, 2011; Sagar et al., 1995). Second, immune activation leads to an increase of circulating corticosterone levels indicating a stimulation of the hypothalamic–pituitary–adrenal (HPA) axis (Lenczowski et al., 1997). Third, the tryptophan catabolite kynurenine, which is generated by indoleamine-2,3-dioxygenase (IDO) upon activation by cytokines, has emerged as a key mediator for the induction of anhedonic and anxiety-like behavior (Haroon et al., 2012; O’connor et al., 2009; Salazar et al., 2012).

Among the more than 20 members of the intracellular NLR family, NOD1 and NOD2 are two widely studied receptors recognizing γ-d-glutamyl-meso-diaminopimelic acid (iE-DAP) and muramyl dipeptide (MDP), respectively (Elinav et al., 2011). Both ligands are produced during the synthesis or degradation of peptidoglycan, with MDP being found in Gram-negative and Gram-positive bacteria, while iE-DAP is predominantly found on Gram-negative bacteria (Chamaillard et al., 2003; Grimes et al., 2012; Mo et al., 2012). NOD1 can also be activated by the synthetic agonist FK565 (Watanabe et al., 1985). Similar to the activation of TLR4, NOD1 and NOD2 activation results in NF-κB- and MAP kinase-dependent inflammatory responses (Elinav et al., 2011). Although NOD agonists are less potent in releasing cytokines than LPS, they are able to potentiate cytokine release induced by LPS challenge in innate immune cells (Le Contel et al., 1993; Netea et al., 2005; Wang et al., 2001; Wolfert et al., 2002). The synergistic induction of cytokine production can also be observed *in vivo* extending to endotoxin shock, with profound hypothermia as one of its hallmarks (Krakauer et al., 2010; Takada and Galanos, 1987).

While there are some reports that MDP induces sleep and anorexia (Fosset et al., 2003; Johannsen et al., 1990; Von Meyenburg et al., 2004), the impact of NOD1 and NOD2 activation on behavior and related brain function has been little studied. Likewise, it is largely unknown whether the interaction of NOD1 and NOD2 stimulation with the TLR4 agonist LPS at the immune level has a bearing on behavior and cerebral activity (Mccusker and Kelley, 2013).

Since in infection both NLRs and TLRs may be activated in parallel, it was the primary aim of the present study to examine the effects of NOD1 and NOD2 activation, alone and in combination with the TLR4 agonist LPS, on sickness, behavior and cerebral c-Fos expression in order to visualize some of the brain nuclei relevant to sickness. The secondary objective was to analyze potential mechanisms behind the synergistic effects of NOD1, NOD2 and TLR4 activation on sickness and behavior. To this end, the effects of NOD1, NOD2 and TLR4 activation on inflammatory indices such as peripheral and central cytokine production and plasma kynurenine/tryptophan ratio were characterized. In addition, HPA axis activation was assessed by measuring circulating corticosterone levels.

## 2 Materials and methods

### 2.1 Experimental animals

The study was carried out with male C57BL/6N mice from Charles River Laboratories (Sulzfeld, Germany) at the age of 10 weeks. The animals were either kept in groups of 2 or singly housed in the institutional animal house. Light conditions (lights on at 6:00 h, lights off at 18:00 h), temperature (set point 22 °C) and relative air humidity (set point 50%) were tightly controlled. Standard laboratory chow and tap water were provided ad libitum throughout the study.

### 2.2 Ethics statement

The experimental procedures and number of animals used were approved by an ethical committee at the Federal Ministry of Science and Research of the Republic of Austria (BMWF-66.010/0119-II/3b/2011) and conducted according to the Directive of the European Communities Council of 24 November 1986 (86/609/EEC). The experiments were designed in such a way that the number of animals used and their suffering was minimized.

### 2.3 Reagents

The chemically synthesized NOD1 agonist FK565 was provided by Astellas Pharma Inc. (Ibaraki, Japan) (Watanabe et al., 1985). MDP (N-acetylmuramyl-l-alanyl-d-isoglutamine hydrate, catalogue number A9519, Sigma–Aldrich, Vienna, Austria) was used as synthetic NOD2 agonist and LPS extracted from *Escherichia* *coli* 0127:B8 (purified by gel-filtration chromatography, catalogue number L3137, Sigma–Aldrich, Vienna, Austria) was used as a TLR4 agonist.

### 2.4 Behavioral testing

The experiments were started after the animals had become accustomed to the institutional animal house over the course of at least 2 weeks. Prior to the behavioral tests, the mice were allowed to adapt to the test room (lights on at 6:00 h, lights off at 18:00 h, set points 22 °C and 50% relative air humidity, maximal light intensity 100 lux) for at least one day.

#### 2.4.1 LabMaster system + sucrose preference

The pattern of locomotion, exploration, feeding as well as sucrose preference (SP) were assessed with the LabMaster system (TSE Systems, Bad Homburg, Germany), allowing continuous recording of the animals without intervention by any investigator, as described previously (Painsipp et al., 2013). The LabMaster system consisted of test cages (type III, 42.0 × 26.5 × 15.0 cm, length × width × height), surrounded by two external infrared frames and a cage lid equipped with three weight transducers. For recording locomotion and exploration, the two external infrared frames were positioned in a horizontal manner above one another at a distance of 4.3 cm, with the lower frame being fixed 2.0 cm above the bedding floor. The bottom frame was used to record horizontal locomotion of the mice, whereas the top frame served to record vertical movements (rearing, exploration). The measures of activity (locomotion, exploration) were derived from the light beam interruptions (counts) of the corresponding infrared frames (Painsipp et al., 2013). The three weight transducers were employed to quantify ingestive behavior. To this end, a feeding bin was filled with standard rodent chow (altromin 1324 FORTI, Altromin, Lage, Germany). In order to assess SP, one drinking bottle was filled with tap water and one with a 1% sucrose solution and the bottles were each attached to a transducer on the cage lid for the total duration of the experiment. SP was calculated using the formula: sucrose intake/(sucrose intake + water intake). In a few cases in which the fluid bottles got obstructed, the data were excluded from analysis.

Each test parameter was collected over a 24 h interval and activity scores and food intake recorded during the day before injection were set as 100%, and the daily scores measured post-injection expressed as a percentage of the pre-injection score.

#### 2.4.2 Open field (OF) test

The OF consisted of a box (50 × 50 × 30 cm) made of opaque gray plastic and was illuminated by 35 lux at floor level (Painsipp et al., 2013). The ground area of the box was divided into a 36 × 36 cm central area and the surrounding border zone. Mice were individually placed in the center of the OF, and their behavior during a 5 min test period was tracked by a video camera positioned above the center of the OF and recorded with the software VideoMot2 (TSE Systems).

#### 2.4.3 Forced swim test (FST)

Mice were individually placed in glass beakers (inner diameter 18 cm, height 27 cm, capacity 5 l) containing tap water at 25 °C (Painsipp et al., 2011). The water depth was 20 cm, which prevented the mice from touching the bottom of the beaker with their paws or the tail. Mice were tested for 6 min and the time of immobility, swimming and climbing was scored by a trained observer blind to the treatment. Mice were considered immobile when floating passively in the water, performing only those movements required to keep their heads above the water level (Cryan et al., 2002).

#### 2.4.4 Tail suspension test (TST)

Mice were suspended by their tail with a 1.9 cm wide strapping tape (Leukotape classic; BSN Medical S.A.S., Le Mans, France) to a lever for 6 min, and their behavior was recorded by a video camera. A trained blinded observer analyzed the video recordings with the VideoMot2 software (TSE Systems) event monitoring module for 3 types of behavior: swinging, curling and immobility. The mouse was considered swinging when it continuously moved its paws while keeping the body straight and/or moving the body from side to side. The mouse was considered curling when the mouse twisted its trunk (Berrocoso et al., 2013). The time spent swinging, curling and being immobile was calculated. Mice which climbed over their tails were excluded as they had learnt that escape is possible (Cryan et al., 2005).

### 2.5 Body temperature

The temperature of the mice was measured with a digital thermometer (BAT-12, Physitemp Instruments, Clifton, New Jersey, USA) equipped with a rectal probe for mice. The temperature recordings were taken between 16:00 and 17:00 h.

### 2.6 Experimental protocols

**Fig. 1:**
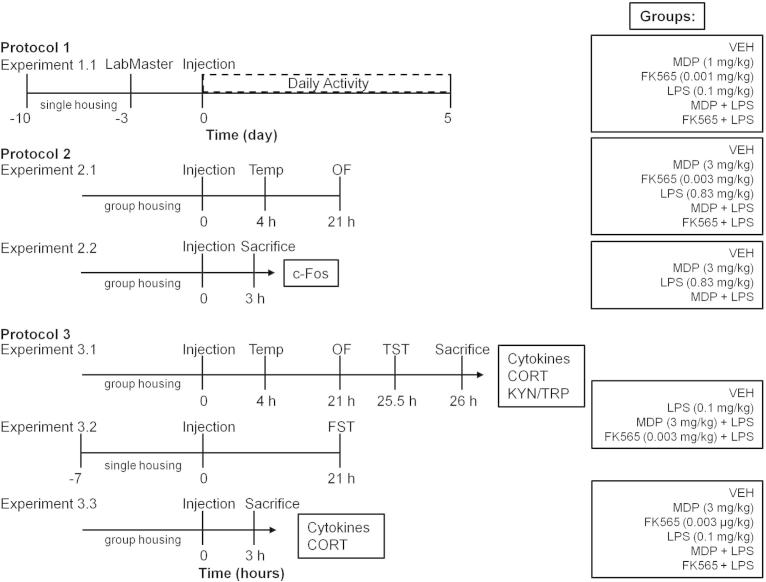
Experimental groups and time lines. In protocol 1, the effects of the PRR agonists on daily activity were assessed in the LabMaster system. In protocol 2, the effects of MDP and FK565 alone and the combination of MDP or FK565 with the higher dose of LPS (0.83 mg/kg) on sickness and central c-Fos expression were evaluated. In protocol 3, the effects of MDP and FK565 alone and the combination of MDP or FK565 with the lower dose of LPS (0.1 mg/kg) on sickness, mood and inflammation-related parameters were analyzed. Time zero represents the time of injection and was consistent across all experiments. Abbreviations: CORT = corticosterone, KYN/TRP = kynurenine/tryptophan ratio, FST = forced swim test, OF = open field, Temp = temperature, TST = tail suspension test.

Three different protocols were used (Fig. 1). For details on the choice of dosing and timing of injections see Sections 2.7 “Dosing” and 2.8 “Timing of injections”.

In protocol 1 (experiment 1.1), the LabMaster system (TSE Systems) was employed to analyze the effects of MDP (1 mg/kg), FK565 (0.001 mg/kg), LPS (0.1 mg/kg), MDP + LPS and FK565 + LPS on the daily pattern of locomotion, exploration, feeding and SP in singly housed mice (Painsipp et al., 2013). The animals were habituated to the drinking bottles used in the LabMaster system and to single housing for 7 days before placing them in the cages of the LabMaster system (Fig. 1). Another 3 days of habituation were warranted in the test cages of the LabMaster system before injection of PRR agonists (*n* = 8).

Protocol 2 was used to carry out 2 separate experiments (Fig. 1). Experiment 1 of protocol 2 (experiment 2.1) was designed to investigate the effects of MDP (3 mg/kg), FK565 (0.003 mg/kg), and the frequently used dose of LPS (0.83 mg/kg), MDP + LPS and FK565 + LPS on the sickness response. For this purpose body temperature and weight were measured immediately before treatment and body temperature was measured again 4 h post-injection. An additional measurement of body weight was taken 21 h post-injection after the animals had been subjected to the open field (OF) test (*n* = 8). In a separate experiment (experiment 2.2), mice were euthanized 3 h after injection of PRR agonists (Fig. 1) and the brains were collected for immunohistochemical visualization of c-Fos expression in select brain regions (*n* = 3–5). Following euthanasia the brains were removed, put on dry ice and stored at −70 °C until use.

Protocol 3 was used in 3 separate experiments (Fig. 1) in which the effects of MDP and FK565 in combination with the lower dose of LPS (0.1 mg/kg) were investigated. In experiment 3.1 body temperature and weight were measured before treatment and the body temperature was measured again 4 h post-injection. The OF test was conducted 21 h after the treatment and body weight was measured after the OF test (Fig. 1). Subsequently the animals were subjected to the tail suspension test (TST) for 6 min (25.5 h post-injection) and euthanized 30 min after start of the TST. Blood was sampled to measure the plasma levels of cytokines, corticosterone, kynurenine and tryptophan (Fig. 1). In addition, the brains were collected, frozen in −70 °C cold 2-methyl butane (Fisher Scientific, Leicestershire, UK) and stored at this temperature until measurement of cytokines (*n* = 7–8).

In experiment 3.2 mice (Fig. 1) were euthanized 3 h after injection of PRR agonists to record the levels of circulating and brain cytokines and circulating corticosterone without interference by any behavioral test (*n* = 7–8).

In a further experiment (experiment 3.3) singly housed mice were subjected to the forced swim test (FST) 21 h post-injection, since depression-like behavior has been shown to be modified by different housing conditions (Painsipp et al., 2011) (*n* = 7–8).

### 2.7 Dosing

All compounds were dissolved in pyrogen-free sterile saline (0.9% NaCl) and pyrogen-free sterile saline injected intraperitoneally (i.p.) at the same volume (50 μL/10 g body weight) was used as vehicle (VEH) control.

For the analysis of the interaction between NOD agonists and LPS, two doses of LPS were examined. First, the widely used dose of 0.83 mg/kg LPS inducing the full spectrum of sickness (Frenois et al., 2007; Painsipp et al., 2011) was used. Since, in combination with the NOD agonists, this dose of LPS led to a marked decrease in body temperature and locomotion, while a ceiling effect was observed with other parameters, a lower dose of 0.1 mg/kg LPS was also tested.

The doses of the NOD agonists (see below) were chosen on the basis of their immunological effects *in vivo* (Parant et al., 1995; Shikama et al., 2011) and the results of pilot experiments. Thus, doses of 1 mg/kg (LabMaster studies) and 3 mg/kg (ex LabMaster studies) of MDP and 0.001 mg/kg (LabMaster studies) and 0.003 mg/kg (ex LabMaster studies) of FK565 were used in order to minimize adverse event rates when combined with the 0.83 mg/kg dose of LPS. Lower doses of the PRR agonists were administered in the LabMaster system because pilot experiments had shown that mice kept singly in the LabMaster system were more sensitive to the treatments than group-housed animals.

### 2.8 Timing of injections

Mice receiving just one of the compounds (MDP, FK565 or LPS) were first injected with saline followed by the respective compound 4 h later. The first injection was given 3 h after start of the light phase. In experiments involving combination treatments, MDP + LPS and FK565 + LPS were given with a time lag of 4 h between injection of the NOD and TLR agonist, since this timing has been shown to have the strongest priming effect on the immune system (Takada et al., 1990; Takada and Galanos, 1987).

Sickness responses were examined 3–4 h after injection and depression-like behavior 21–26 h post-treatment. The time points for the recording of the sickness responses were chosen according to the known time course of the sickness response to MDP or LPS (Frenois et al., 2007; Engeland et al., 2003). Sickness behavior has been shown to pass into depression-like behavior 1 day after injection of LPS (0.83 mg/kg), which was the reason for choosing the second time point (Frenois et al., 2007). Since MDP or FK565 alone did not induce behavioral changes in experiment 2.1 and only induced a modest cytokine response 3 h post-treatment, the single treatment with MDP or KF565 was not investigated in experiments 3.1 and 3.2.

### 2.9 Blood sampling

Mice were deeply anesthetized with pentobarbital (150 mg/kg i.p.). Blood was sampled by cardiac puncture using citrate (3.8%) as an anticoagulant. Following centrifugation for 10 min at 4 °C and 1000×*g*, blood plasma was collected and stored at −70 °C until assay.

### 2.10 Circulating corticosterone

The plasma levels of corticosterone were determined with an enzyme immunoassay kit (Assay Designs, Ann Arbor, Michigan, USA). According to the manufacturer’s specifications, the sensitivity of the assay is 27 pg/mL, and the intra- and inter-assay coefficient of variation amounts to 7.7% and 9.7%, respectively.

### 2.11 Kynurenine and tryptophan

Kynurenine and tryptophan were measured in plasma samples by high-performance liquid chromatography (HPLC) with ultraviolet detection (Herve et al., 1996). In brief, 100 μL plasma samples were deproteinized by adding of 100 μL of 5% (v/v) perchloric acid. After vortexing and 5 min centrifugation at 11,000×*g*, 20 μL of the clear supernatant was injected in the chromatographic system. Separations were achieved on a Chromolith RP18e column (100 × 4.6 mm, 5 μm, Merck Darmstadt, Germany) at 30 °C by isocratic elution with a mobile phase consisting of 50 mmol/L ammonium acetate, 250 mol/L zinc acetate and 3% (v/v) acetonitrile (pH 4.9) at a flow rate of 0.8 mL/min. Kynurenine and tryptophan were detected on a LaChrom UV-Detector Merck HITACHI L-7400 at 235 nm. Acquisition and processing of the chromatograms were performed using a Merck Hitachi LaChrom®-D-7000 HPLC-System Manager software (VWR International GmbH/Scientific Instruments, Darmstadt, Germany). The concentrations were determined by peak-height measurement against external standards. This method has been validated according to international guidelines (Center for Veterinary Medicine (CVM), 2001). All reagents were of p.A. grade (Merck, Darmstadt, Germany). The within-day coefficient of variation (CV) at different concentrations ranged from 1.7% to 4.3%, for kynurenine and 0.7% to 2.9% for tryptophan. The between day CVs were 2.0–5.4% and 6.3–9.3% respectively.

### 2.12 Circulating cytokines

Concentrations of IFN-γ, IL-1β, IL-6 and TNF-α were simultaneously quantified in plasma using the ProcartaPlex™ immunoassay (eBioscience, San Diego, CA, USA). Cytokine concentrations were determined using analyte specific capture beads coated with target-specific capture antibodies according to the manufacturer’s specifications. The analytes were detected by biotinylated analyte-specific antibodies. Following binding of the fluorescent detection label (SA-PE), the reporter fluorescent signal was measured with the Bio-Plex 200 multiplex suspension array system employing Luminex xMAP technology in combination with the Bio-Plex 5.0 Software (Bio-Rad, Hercules, CA).

Standard curves for each analyte were generated by using the reference analyte concentration supplied and concentrations were calculated using a five-parameter logistic curve-fitting method. Cytokines that were not detected were assigned a value of zero. The sensitivity for the respective cytokines was: IFN-γ: 0.09 pg/mL, IL-1β: 0.14 pg/mL, IL-6: 0.21 pg/mL, TNF-α: 0.39 pg/mL.

Plasma samples of experiment 3.3 were run in duplicate. Since the coefficient of variance for the duplicate samples was small, single samples were run subsequently.

### 2.13 RNA extraction and reverse transcription

LPS has been reported to induce a ubiquitous upregulation of cytokine mRNA expression in discrete brain regions (O’connor et al., 2009). Thus, one part of a hemibrain (Bregma +0.50 to −2.70) weighing 50–60 mg was dissected on a cold plate and homogenized in MagnaLyser bead tubes (Catalogue number 03358 941 001, Roche Diagnostics, Rotkreuz, CH) using the MagnaLyser centrifuge (Roche Diagnostics). Total RNA was extracted in TRIzol reagent (Catalogue number 15596018, Life Technologies, Carlsbad, CA) and randomly tested for quality on the BioAnalyzer BA2100 (Agilent, Foster City, CA) with the RNA 6000 Nano LabChip Kit (Catalogue number 5067-1511, Agilent, Foster City, CA). The RIN (RNA Integrity Number) of all tested samples ranged between 7.9 and 8.7. All RNA samples were reverse transcribed simultaneously in the Thermocycler ‘MyCycler’ (Bio-Rad Laboratories, Hercules, CA), using the High Capacity cDNA Reverse Transcription Kit (Catalogue number 4368813, Life Technologies) according to manufacturer instructions.

#### 2.13.1 Real-time RT-PCR

Real-time RT-PCR was performed on a LightCycler®480 System using TaqMan® gene expression assays for TNF-α (Mm00443258_m1), IL-1β (Mm00434228_m1), IFN-γ (Mm01168134_m1), IL-6 (Mm00446190_m1), Gapdh (Mm999999_g1) and Actb (Mm00607939_s1) purchased from Life Technologies (Catalogue number 4331182). For the real-time RT-PCR setup the TaqMan Gene Expression Master Mix (Catalogue number 4369016, Life Technologies) was used. Reactions were carried out in triplicates according to manufacturer instructions using a 20 ng cDNA template for each reaction. As negative controls, amplifications without reverse transcription or template were included. Quantitative measurement of target gene levels relative to controls was performed with the 2^−ΔΔCt^ method (Schmittgen and Livak, 2008). Gapdh and Actb were used as endogenous housekeeping genes.

### 2.14 c-Fos immunohistochemistry

The activation of neurons in select nuclei and cortical areas of the brain was visualized by c-Fos immunohistochemistry 3 h after injection of PRR agonists. Immunohistochemistry was performed according to a slightly modified version of the protocol provided by Sundquist and Nisenbaum (2005) and described by Reichmann et al. (2013). The primary antibody used was rabbit polyclonal anti-c-Fos SC-52 (Santa Cruz Biotech, Santa Cruz, California, USA, 1:2000 dilution). As the secondary antibody, the biotinylated goat anti-rabbit IgG (Vectastain Elite ABC Kit, Vector Laboratories, 1:200 dilution) was used. The sections were incubated in avidin–biotin complex (Vectastain Elite ABC Kit, Vector Laboratories) and developed with 3,3-diaminobenzidine substrate (DAB substrate kit for peroxidase, Vector Laboratories).

#### 2.14.1 Cell counting and quantification

The immunohistochemically processed brain sections were examined with a light microscope (Axiophot, Zeiss, Oberkochen, Germany) coupled to a computerized image analysis system (MCID Basic, version 7.0, Imaging Research Inc., Brock University, St. Catharines, Ontario, Canada) as described previously (Reichmann et al., 2013). While in the paraventricular nucleus of the hypothalamus (PVN) and the granular cell layer of the dentate gyrus all c-Fos positive cells were counted, the number of c-Fos labeled cells in the other regions of interest (ROIs) was quantitated within a square of 200 × 200 μm, and the c-Fos labeled cells of the subfornical organ were quantitated within a square of 400 × 400 μm. One section was counted bilaterally to quantitate the number of c-Fos positive cells in the dorsal part of the bed nucleus of the stria terminalis (BNSTd) (Bregma +0.38 to +0.14), while two consecutive sections were counted bilaterally to quantitate the number of c-Fos positive cells in the ventral part of the bed nucleus of the stria terminalis (BNSTv) (Bregma +0.50 to +0.14), the paraventricular nucleus of the hypothalamus (PVN) (Bregma −0.58 to −0.94), the insula (Bregma +0.38 to +0.14), and the subfornical organ (SFO) (Bregma −0.58 to −0.70). Three consecutive sections were counted bilaterally to quantitate the number of c-Fos positive cells in the central amygdala (CeA) (Bregma −1.34 to −1.70), the supraoptic nucleus (SO) (Bregma −0.70 to −1.06), and the dentate gyrus (DG) (Bregma −1.34 to −1.94). The cell counts obtained for each ROI in the different sections of each animal were averaged to calculate the mean number of c-Fos-positive cells within a particular brain region of that animal. These average values/brain region of each animal were used for statistical analysis.

### 2.15 Statistics

Statistical evaluation of the results was made with SPSS 20 (SPSS Inc., Chicago, Illinois, USA). In general, the data were analyzed by one-way or two-way analysis of variance (ANOVA), as appropriate, in some cases for repeated measurements. Two-way ANOVA was performed with the NOD agonists (VEH, MDP, FK565) and LPS (VEH, LPS) as the between subject variables in order to reveal significant main factor effects or interactions denoted as NOD × LPS interactions. The homogeneity of variances was assessed with the Levene test. In case of sphericity violations the Greenhouse–Geisser correction was applied. Post-ANOVA analysis of group differences was performed with the Tukey HSD (honestly significant difference) test, when the variances were homogeneous, and with the Games–Howell test, when the variances were unequal. In case of a non-parametric distribution of the parameters, statistical differences among groups were determined with the Kruskal–Wallis test and post-hoc analysis of group differences was performed with the Mann–Whitney test. *p* values were adjusted for multiple comparisons with the Bonferroni correction. Probability values of *p* < 0.05 were regarded as statistically significant and *p* < 0.1 were regarded as a trend. All data are presented as means + SEM, n referring to the number of mice in each group.

## 3 Results

### 3.1 Effects of MDP, FK565 and LPS on daily levels of locomotion, exploration, food intake and SP

**Fig. 2:**
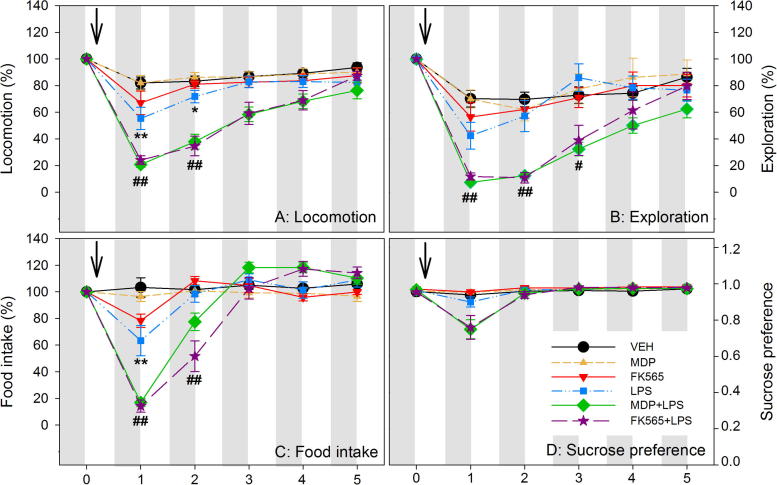
Effects of MDP, FK565 and LPS on daily levels of locomotion (A), exploration (B), food intake (C) and sucrose preference (D) in male mice. Saline (VEH), MDP (1 mg/kg), FK565 (0.001 mg/kg), LPS (0.1 mg/kg), MDP + LPS or FK565 + LPS were injected i.p. as indicated. The graphs show the daily levels of activity for 1 day before (set as 100% in A, B and C) and 5 days after injection. The values are means ± SEM, *n* = 8. Post-hoc analysis of significant NOD × LPS interactions in 2-way ANOVA: ^∗^*p* < 0.05, ^∗∗^*p* < 0.01, versus VEH. ^#^*p* < 0.05, ^##^*p* < 0.01, MDP + LPS or FK565 + LPS versus MDP, FK565 or LPS.

MDP, FK565 and LPS altered locomotion, exploration, food intake and SP in a compound-, combination- and time-dependent manner (Fig. 2). Repeated measures ANOVA revealed a significant interaction of NOD (VEH, MDP, FK565) × LPS (VEH, LPS) × time (days post-treatment) for the variation in locomotion (*F*_(5.661,116.05)_ = 2.457, *p* < 0.05). The same was true for exploratory behavior (*F*_(5.250,110.25)_ = 2.470, *p* < 0.05). Likewise, there was a significant NOD × LPS × time interaction for the differences in food intake (*F*_(5.025,105.52)_ = 5.244, *p* < 0.001). SP depended on time (*F*_(1.130,39.55)_ = 27.838, *p* < 0.001), with a significant interaction with LPS (*F*_(1.130,39.55)_ = 18.397, *p* < 0.001) and an interaction with the NOD agonists by trend (*F*_(2.260,39.55)_ = 2.339, *p* = 0.10).

Post-hoc analysis revealed significant NOD × LPS interactions on day 1 and 2 post-treatment. While MDP (1 mg/kg) and FK565 (0.001 mg/kg) alone did not induce any significant changes in locomotion, LPS (0.1 mg/kg) led to a decrease of locomotion for 2 days after injection when compared with the VEH-treated group. Combination of MDP + LPS attenuated locomotion compared to treatment with MDP or LPS alone during day 1 and 2 post-treatment (Fig. 2A). Likewise, the combination of FK565 + LPS significantly decreased locomotion when compared with FK565 or LPS alone. Post-hoc analysis of the changes in exploration disclosed a significant NOD × LPS interaction on day 1 and 3 post-treatment, while there was only a trend for interaction on day 2 (*p* = 0.1). Specifically, MDP + LPS and FK565 + LPS decreased exploration when compared with LPS or MDP and FK565, respectively (Fig. 2B).

A significant NOD × LPS interaction was evident for food intake on day 1 and 2 post-treatment (Fig. 2C). While the effect of FK565 did not reach statistical significance after correcting for multiple testing, LPS diminished food intake 1 day after treatment when compared to VEH. Again, MDP + LPS and FK565 + LPS further attenuated food intake 1 day post-treatment compared to MDP and FK565, respectively. Both combinations also led to a decrease of food intake when compared with LPS (Fig. 2C). On day 2 post-treatment food intake was still decreased in the FK565 + LPS group compared to the FK565 or LPS groups, while the effect of MDP + LPS did not reach significance after correcting for multiple testing.

Unlike LPS, MDP + LPS or FK565 + LPS led to a nominal decline of SP on day 1 post-treatment, but the interaction of LPS with the NOD agonists did not reach statistical significance (Fig. 2D).

### 3.2 Effects of MDP and FK565 alone and in combination with LPS on body temperature and weight

**Fig. 3:**
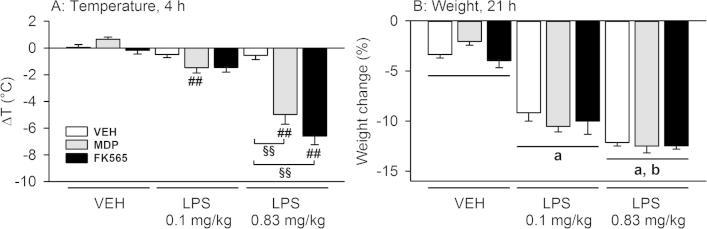
Effects of MDP (3 mg/kg), FK565 (0.003 mg/kg) and LPS (doses as indicated) to reduce body temperature (A) and weight (B) in male mice. The graphs show the change in temperature 4 h post-treatment and body weight 21 h post-treatment. Body temperature and weight were measured before treatment and 4 and 26 h post-injection, respectively. The weight loss induced by the treatment is expressed as a percentage of the body weight measured pre-treatment. The values are means + SEM, *n* = 15 for VEH (merged from 2 separate experiments), *n* = 7–8 for other groups. Post-hoc analysis of significant NOD × LPS interactions in 2-way ANOVA: ^##^*p* < 0.01, MDP + LPS versus MDP or FK565 + LPS versus FK565. ^§§^*p* < 0.01, MDP + LPS and FK565 + LPS versus LPS (0.83 mg/kg). Main factor effects without NOD × LPS interactions: ^a^*p* < 0.01, LPS versus VEH. ^b^*p* < 0.01, LPS 0.83 mg/kg versus LPS 0.1 mg/kg.

MDP, FK565 and LPS interacted with each other in modifying body temperature but not body weight (Fig. 3). Two-way ANOVA revealed a significant NOD × LPS interaction for the changes in body temperature (*F*_(4,65)_ = 20.413, *p* < 0.001) (Fig. 3A). Post-hoc analysis showed that neither MDP (3 mg/kg), FK565 (0.003 mg/kg) nor the two doses of LPS induced changes of body temperature 4 h post-treatment. In contrast, combined treatment with MDP + LPS (0.83 mg/kg) and FK565 + LPS (0.83 mg/kg) evoked a strong hypothermic response compared to single treatment with the NOD agonists or LPS (Fig. 3A). Also the combination of MDP or FK565 with the lower dose of LPS (0.1 mg/kg) slightly decreased body temperature, the effect of MDP + LPS (0.1 mg/kg) reaching statistical significance when compared to MDP alone (Fig. 3A).

The effects on body weight differed from those on body temperature. Thus, a NOD × LPS interaction was not evident for the differences in weight (Fig. 3B). Two-way ANOVA showed that weight loss depended solely on LPS (*F*_(2,67)_ = 166.200, *p* < 0.001) (Fig. 3B).

### 3.3 Effects of MDP and FK565 alone and in combination with LPS on behavior in the OF

**Fig. 4:**
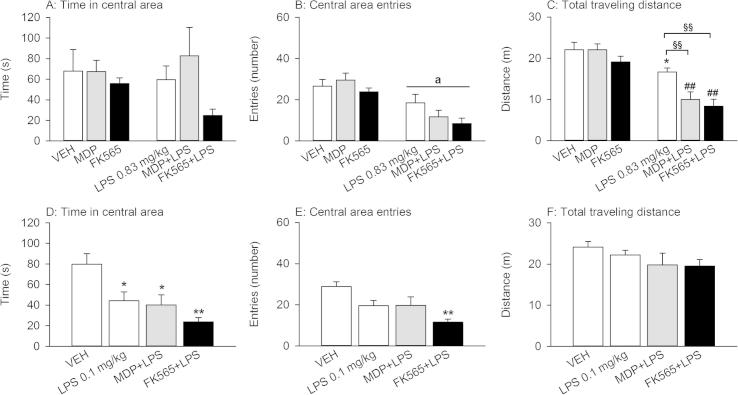
Effects of MDP (3 mg/kg), FK565 (0.003 mg/kg) and LPS (doses as indicated) on behavior in the OF 21 h post-treatment in male mice. The graphs show the time spent in the central area (A + D), the number of entries into the central area (B + E) and the total distance traveled (C + F) during the 5 min test session. The values are means + SEM, *n* = 7–8. (A–C): Post-hoc analysis of significant NOD × LPS interactions in 2-way ANOVA: ^∗^*p* < 0.05, versus VEH. ^##^*p* < 0.01, MDP + LPS versus MDP or FK565 + LPS versus FK565. ^§§^*p* < 0.01, MDP + LPS or FK565 + LPS versus LPS. Main factor effects without NOD × LPS interactions: ^a^*p* < 0.01, LPS versus VEH. (D–F): One-way ANOVA: ^∗^*p* < 0.05, ^∗∗^*p* < 0.01, versus VEH.

The behavior in the OF was modified by MDP, FK565 and LPS in a compound-, combination- and time-dependent manner (Fig. 4). The OF test was used to assess anxiety-like behavior as deduced from the time spent in the central area and the entries made to the central area of the OF and locomotion as deduced from the traveling distance (Fig. 4). In experiments with the higher dose of LPS (0.83 mg/kg), two-way ANOVA revealed a significant NOD × LPS interaction for the changes in locomotion (*F*_(2,42)_ = 3.168, *p* ⩽ 0.05). Post-hoc analysis showed that while the NOD agonists did not impact on locomotion, treatment with LPS (0.83 mg/kg) slightly decreased the traveling distance in the OF (Fig. 4C). Furthermore, combined treatment with MDP (3 mg/kg) + LPS (0.83 mg/kg) or FK565 (0.003 mg/kg) + LPS (0.83 mg/kg) further diminished the distance traveled when compared with LPS alone, or MDP and FK565, respectively (Fig. 4C). The entries made into the center of the field depended on LPS (*F*_(1,42)_ = 31.001, *p* < 0.001), while the effect of the NOD agonists and their interaction with LPS did not reach significance (Fig. 4B). The time spent in the central area of the OF was not significantly affected by any of the compounds (Fig. 4A).

In experiments with the lower dose of LPS (0.1 mg/kg), LPS alone, MDP + LPS (0.1 mg/kg), as well as FK565 + LPS (0.1 mg/kg) reduced the time spent in the central area of the field (Fig. 4D) and the entries made to the central area (Fig. 4E) without affecting the total distance traveled (Fig. 4F). The combination of FK565 + LPS had the most pronounced effects. While the time in the central area was reduced in all groups (*F*_(3,25)_ = 7.176, *p* = 0.001) (Fig. 4D), the entries made to the central area of the field were solely reduced by FK565 + LPS (*F*_(3,25)_ = 6.256, *p* < 0.01) (Fig. 4E).

### 3.4 Effects of MDP, FK565 and LPS alone and in combination on depression-like behavior in the FST and TST

LPS (0.1 mg/kg) did not change any behavioral parameter in the FST. In contrast, combined treatment with MDP + LPS and FK565 + LPS induced a slight increase of immobility and a decrease of the duration of time spent swimming, but these changes did not reach statistical significance (Table 1). Likewise, in the TST there were no significant changes in the duration of immobility, swinging or curling by any of the treatments (Table 1).

### 3.5 Effects of MDP, FK565 and LPS alone and in combination on circulating cytokines

**Fig. 5:**
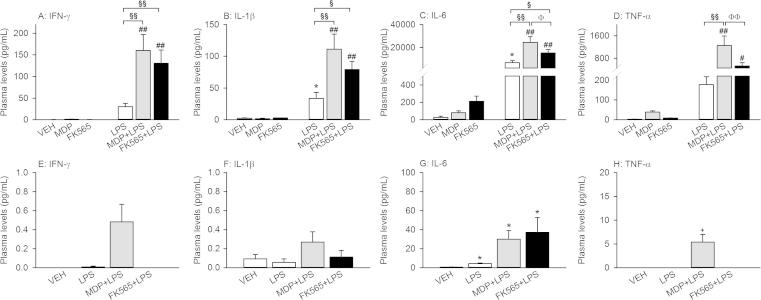
Effects of MDP (3 mg/kg), FK565 (0.003 mg/kg) and LPS (0.1 mg/kg) on circulating cytokine levels 3 h (A–D) and 26 h (E–H) after treatment of male mice. Mice were injected i.p. with saline (VEH), MDP, FK565, LPS, MDP + LPS or FK565 + LPS. Plasma was collected 3 h (A–D) or 26 h (E–H) later. Circulating levels of IFN-γ (A + E), IL-1β (B + F), IL-6 (C + G), and TNF-α (D + H) were measured. Note that the scale of the ordinate is different between the 2 time points. The values are means + SEM, *n* = 7–8. (A–D): Post-hoc analysis of significant NOD × LPS interactions in 2-way ANOVA: ^∗^*p* < 0.05, versus VEH. ^#^*p* < 0.05, ^##^*p* < 0.01, MDP + LPS versus MDP or FK565 + LPS versus FK565. ^§^*p* < 0.05, ^§§^*p* < 0.01, MDP + LPS or FK565 + LPS versus LPS. ^Φ^*p* < 0.05, ^ΦΦ^*p* < 0.01, MDP + LPS versus FK565 + LPS. (E–H): One-way ANOVA: ^∗^*p* < 0.05, ^∗∗^*p* < 0.01, versus VEH.

MDP, FK565 and LPS, alone and in combination, had distinct effects to enhance the circulating levels of proinflammatory cytokines (Fig. 5). Three hours after injection, there was a significant NOD × LPS interaction with regard to the circulating levels of IFN-γ (*F*_(2,39)_ = 6.004, *p* < 0.01), IL-1β (*F*_(2,40)_ = 6.274, *p* < 0.01), IL-6 (*F*_(2,40)_ = 7.092, *p* < 0.01) and TNF-α (*F*_(2,40)_ = 7.665, *p* < 0.01) (Fig. 5A–D). Post-hoc analysis revealed that treatment with MDP (3 mg/kg) or FK565 (0.003 mg/kg) alone did not induce significant increases in the plasma levels of the cytokines measured (Fig. 5). LPS (0.1 mg/kg) alone increased circulating IL-1β and IL-6 levels compared to VEH (Fig. 5B and C). In contrast, treatment with MDP or FK565 + LPS increased the levels of all circulating cytokines under study relative to MDP and FK565, respectively (Fig. 5A–D). In addition, the cytokine levels in the MDP + LPS group were significantly higher than in the LPS group and with regard to IL-6 and TNF-α were even larger than in the FK565 + LPS group (Fig. 5C and D). The cytokine levels in the FK565 + LPS group were increased compared to LPS for all measured cytokines except TNF-α.

Twenty-six hours after treatment, the circulating levels of IFN-γ, IL-1β, IL-6 and TNF-α had largely decreased in all groups studied and were below the detection limit in many samples (Fig. 5E–H). As seen 3 h post-treatment, the highest levels of IFN-γ, IL-1β and TNF-α were recorded in the MDP + LPS treatment group, with a trend being evident for TNF-α (*U* = 7.000, *p* = 0.054, Bonferroni correction). IL-6 remained significantly increased in all treatment groups (LPS: *U* = 3.000, *p* = 0.018; Bonferroni correction, MDP + LPS: *U* = 2.000, *p* = 0.018, Bonferroni correction; FK565 + LPS, *U* = 2.000, *p* = 0.012, Bonferroni correction), comparable levels being seen in the MDP + LPS and FK565 + LPS treatment groups (Fig. 5G).

### 3.6 Effects of MDP, FK565 and LPS alone and in combination on cytokine mRNA expression in the brain

**Fig. 6:**
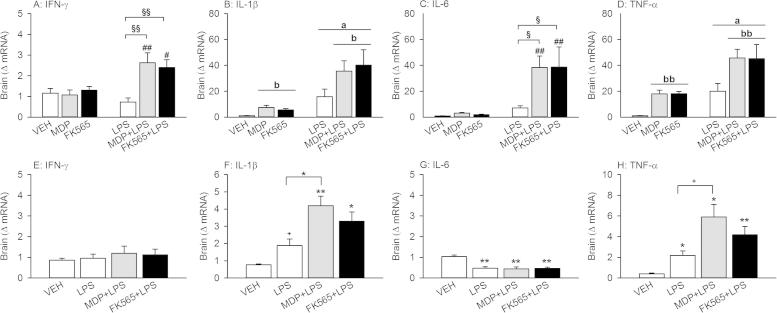
Effects of MDP (3 mg/kg), FK565 (0.003 mg/kg) and LPS (0.1 mg/kg) on cytokine mRNA expression in the brain 3 h (A–D) and 26 h (E–H) after injection in male mice. Mice were injected i.p. with saline (VEH), MDP, FK565, LPS, MDP + LPS or FK565 + LPS. Expression of IFN-γ (A + E), IL-1β (B + F), IL-6 (C + G), and TNF-α (D + H) was measured 3 h (A–D) or 26 h (E–H) after injection. The values are means + SEM, *n* = 7–8. (A–D): Post-hoc analysis of significant NOD × LPS interactions in 2-way ANOVA: ^#^*p* < 0.05, ^##^*p* < 0.01, MDP + LPS versus MDP or FK565 + LPS versus FK565. ^§^*p* < 0.05, ^§§^*p* < 0.01, MDP + LPS and FK565 + LPS versus LPS. Main factor effects without NOD × LPS interactions: ^a^*p* < 0.01, LPS versus VEH. ^b^*p* < 0.05, ^bb^*p* < 0.01, NLR agonists versus VEH. (E–H): One-way ANOVA: ^+^*p* < 0.1, ^∗^*p* < 0.05, ^∗∗^*p* < 0.01, versus VEH or as indicated by the brackets.

The expression of cytokine mRNAs in the brain was measured 3 and 26 h after injection of the PRR agonists in order to analyze cytokine expression at the time of predominant sickness and depression-like behavior, respectively (Fig. 6). When cytokine mRNA was assessed 3 h post-treatment, two-way ANOVA revealed a NOD × LPS interaction for the expression of IFN-γ mRNA (*F*_(2,42)_ = 5.911, *p* < 0.01) and a trend for IL-6 mRNA expression (*F*_(2,42)_ = 2.774, *p* = 0.07). Post-hoc analysis disclosed that while neither MDP (3 mg/kg), FK565 (0.003 mg/kg) nor LPS (0.1 mg/kg) alone increased mRNA expression of IFN-γ or IL-6, combined treatment with MDP + LPS or FK565 + LPS increased IFN-γ and IL-6 mRNA expression compared to LPS or MDP and FK565, respectively (Fig. 6A and C). In contrast, expression of IL-1β mRNA depended on LPS (*F*_(1,42)_ = 24.984, *p* < 0.001) and the NOD agonists (*F*_(2,42)_ = 3.174, *p* ⩽ 0.05) without a significant interaction (Fig. 6B). Likewise, TNF-α mRNA expression depended on LPS (*F*_(1,42)_ = 25.735, *p* < 0.001) and the NOD agonists (*F*_(2,42)_ = 8.535, *p* < 0.001) without a significant interaction (Fig. 6D).

Twenty-six hours after treatment, cerebral IFN-γ mRNA expression had returned to basal levels in all treatment groups (Fig. 6E). Conversely, the expression of IL-1β mRNA remained significantly increased in response to MDP + LPS and FK565 + LPS (*F*_(3,26)_ = 11.341, *p* < 0.001) and enhanced by trend in the LPS group (*p* = 0.085). In addition, IL-1β mRNA expression was significantly higher in the MDP + LPS group compared to the LPS group (Fig. 6F). Likewise, TNF-α mRNA expression was increased in every treatment group (*F*_(3,26)_ = 9.588, *p* < 0.001), with the highest expression seen in the MDP + LPS group (Fig. 6H). In contrast, IL-6 mRNA expression was decreased in all treatment groups (*F*_(3,26)_ = 13.621, *p* < 0.001) (Fig. 6G).

### 3.7 Effects of MDP, FK565 and LPS alone or in combination on circulating levels of corticosterone and the kynurenine/tryptophan ratio

**Fig. 7:**
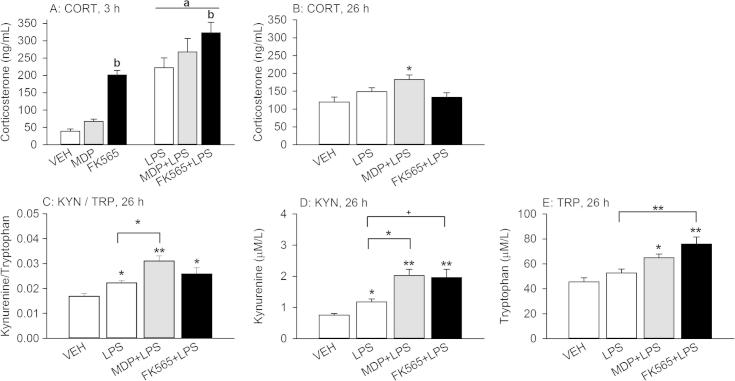
Effects of MDP (3 mg/kg), FK565 (0.003 mg/kg) and LPS (0.1 mg/kg) on circulating corticosterone (CORT) levels 3 h (A) and 26 h (B) after treatment, as well as on the kynurenine/tryptophan (KYN/TRP) ratio (C) and circulating levels of kynurenine (D) and tryptophan (E) measured 26 h after treatment of male mice. Plasma CORT was measured 3 h after treatment (A) and 26 h after treatment, 30 min following exposure to tail suspension stress (B). Likewise, plasma kynurenine and tryptophan (C–E) were determined 26 h after treatment, 30 min following exposure to tail suspension stress. The values are means + SEM, *n* = 7–8. (A): Main factor effects without NOD × LPS interactions: ^a^*p* < 0.001, LPS versus VEH. ^b^*p* < 0.001, FK565 versus VEH or MDP. (B–E): One-way ANOVA: ^+^*p* < 0.1, ^∗^*p* < 0.05, ^∗∗^*p* < 0.01, versus VEH or as indicated by the brackets.

The PRR agonists under study had a distinct effect to enhance the plasma levels of corticosterone as measured 3 h after injection. Two-way ANOVA revealed a significant main factor effect for LPS (*F*_(1,40)_ = 76.581, *p* < 0.001) and the NOD agonists (*F*_(2,40)_ = 16.608, *p* < 0.001) without a significant interaction. Post-hoc analysis of the main factor effects disclosed that FK565 increased circulating corticosterone compared to VEH and MDP (Fig. 7A).

One day after treatment, the plasma levels of corticosterone were examined 30 min after exposure to the TST. Under these conditions, the circulating levels of corticosterone were enhanced in the MDP + LPS treatment group relative to the vehicle (but not LPS) treatment group and remained unchanged in the LPS and FK565 + LPS treatment groups (*F*_(3,26)_ = 4.282, *p* < 0.05) (Fig. 7B).

The plasma kynurenine/tryptophan ratio, measured 26 h after treatment, was significantly increased following injection of LPS, MDP + LPS and FK565 + LPS (*F*_(3,26)_ = 10.160, *p* < 0.001), this increase being more pronounced after treatment with MDP + LPS. Particularly, the plasma kynurenine/tryptophan ratio in the MDP + LPS treatment group was significantly larger than in the LPS-treated group (Fig. 7C).

**Fig. 8:**
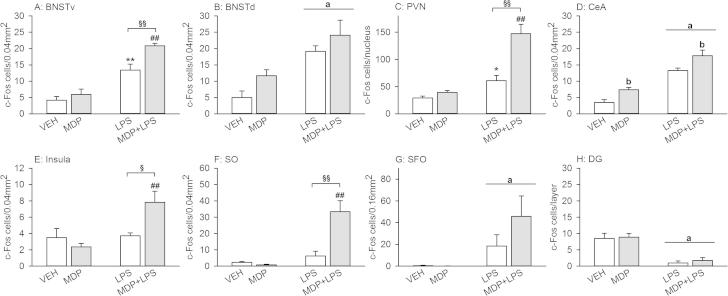
Effects of MDP (3 mg/kg), LPS (0.83 mg/kg) and MDP + LPS on c-Fos expression 3 h after treatment of male mice. Mice were injected i.p. with saline (VEH), MDP, LPS or MDP + LPS. Brains were collected 3 h post-treatment and the expression of c-Fos, a marker of neuronal activation, was measured by immunohistochemistry. The values are means + SEM, *n* = 3–5. Post-hoc analysis of significant NOD × LPS interactions in 2-way ANOVA: ^∗^*p* < 0.05, ^∗∗^*p* < 0.01, versus VEH. ^##^*p* < 0.01, MDP + LPS versus MDP. ^§^*p* < 0.05, ^§§^*p* < 0.01, MDP + LPS versus LPS. Main factor effects without NOD × LPS interactions: ^a^*p* < 0.01, LPS versus VEH. ^b^*p* < 0.01, NLR agonist versus VEH. *Abbreviations:* BNSTd/v = bed nucleus of the stria terminalis dorsal/ventral, CeA = central amygdala, DG = dentate gyrus, PVN = paraventricular nucleus of the hypothalamus, SFO = subfornical organ, SO = supraoptic nucleus.

A similar picture emerged for the circulating levels of kynurenine (Fig. 7D). Kynurenine levels were increased by LPS, MDP + LPS and FK565 + LPS (*F*_(3,26)_ = 12.098, *p* < 0.001). As for the kynurenine/tryptophan ratio, the kynurenine levels in the MDP + LPS group were significantly higher than in the LPS group, while the levels in the FK565 + LPS group were increased by trend only compared to LPS alone (*p* = 0.077). The levels of tryptophan were increased by MDP + LPS and FK565 + LPS, while LPS alone did not change the plasma tryptophan levels (*F*_(3,26)_ = 11.207, *p* < 0.001) (Fig. 8E). Furthermore, the tryptophan levels in the FK565 + LPS group were significantly higher than in the LPS group.

### 3.8 Effects of MDP and LPS (0.83 mg/kg) alone and in combination on c-Fos expression in the brain

In order to analyze brain circuits that are associated with the observed effects of MDP (3 mg/kg) and LPS (0.83 mg/kg), the expression of c-Fos was studied by immunohistochemistry in select brain areas involved in sickness.

Two-way ANOVA revealed a significant NOD × LPS interaction in the PVN (*F*_(1,11)_ = 18.810, *p* < 0.001), insula (*F*_(1,13)_ = 6.940, *p* < 0.05) and SO (*F*_(1,13)_ = 17.496, *p* ⩽ 0.001) and an interaction approaching significance in the BNSTv (*F*_(1,15)_ = 4.257, *p* = 0.057). Post-hoc analysis disclosed that MDP alone did not change c-Fos expression in these areas, while LPS alone increased c-Fos expression in the BNSTv and PVN compared to VEH (Fig. 8A and C). In contrast, MDP + LPS increased c-Fos expression in all 4 areas relative to MDP or LPS (Fig. 8A, C, E and F).

While LPS had a significant main factor effect in all other areas under study (BNSTd: *F*_(1,13)_ = 16.883, *p* < 0.001; CeA: *F*_(1,15)_ = 80.556, *p* < 0.001; SFO: *F*_(1,14)_ = 11.334, *p* < 0.01; DG: *F*_(1,15)_ = 39.727, *p* < 0.001), a significant main factor effect of the NOD agonist MDP was evident in the CeA (*F*_(1,15)_ = 14.296, *p* < 0.01) and by trend in the BNSTd (*F*_(1,13)_ = 3.237, *p* < 0.1) (Fig. 8B, D, G and H). The effect of MDP + LPS to increase the number of c-Fos positive cells in the SFO, relative to LPS, was statistically not significant (Fig. 8G).

**Fig. 9:**
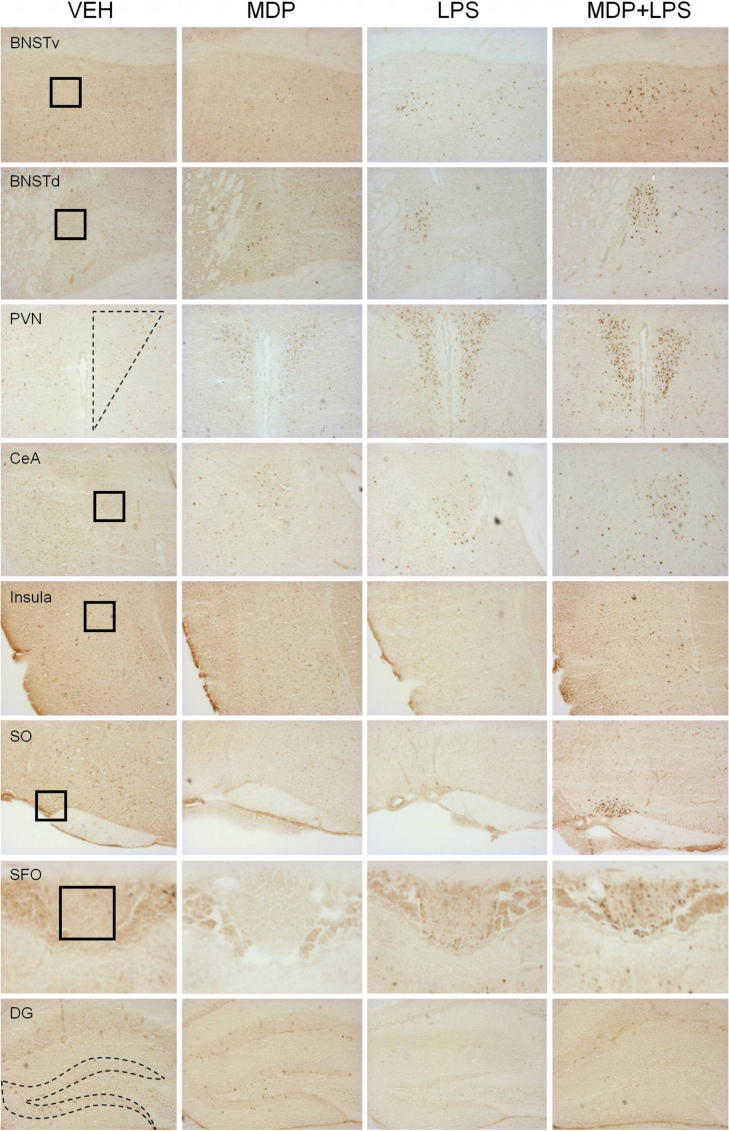
Representative micrographs of forebrain regions illustrating c-Fos immunoreactivity induced by MDP (3 mg/kg), LPS (0.83 mg/kg) and MDP + LPS in male mice. The left column panels show micrographs of forebrain regions taken from saline (VEH)-treated mice euthanized 3 h after injection. The second column panels depict micrographs of the same brain regions taken from MDP-treated mice, while the third column panels show micrographs of LPS-treated mice. The right column panels represent c-Fos immunolabeling induced by MDP + LPS 3 h after treatment. The squares in the left column represent the position and size of the ROIs. *Abbreviations:* BNSTd/v = bed nucleus of the stria terminalis dorsal/ventral, CeA = central amygdala, DG = dentate gyrus, PVN = paraventricular nucleus of the hypothalamus, SFO = subfornical organ, SO = supraoptic nucleus.

Representative micrographs showing the effects of MDP, LPS and MDP + LPS on the expression of c-Fos in the cerebral areas under study are shown in Fig. 9.

## 4 Discussion

This study provides a multivariate assessment of the effects of the NOD1 agonist FK565 and the NOD2 agonist MDP, alone and in combination with the TLR4 agonist LPS, on immune, cerebral, neuroendocrine and behavioral parameters of sickness in male mice. The results revealed that NOD1 and NOD2 activation interacts with TLR4 agonism in inducing sickness. In analyzing the nature of this interaction (additive versus synergistic) it would have been desirable to construct dose–response curves, but such experiments were not deemed acceptable in view of ethical considerations. This disadvantage was balanced by a careful choice of the compound doses under study, based on the available literature and the results of pilot experiments. Thus, the NOD agonist doses were chosen such that they failed to induce sickness by their own, yet were able to enhance the sickness response to LPS. By comparing the effects of the PRR agonists alone with those of FK565 + LPS and MDP + LPS it was disclosed that NOD and TLR4 agonism interacted with each other either in a synergistic or additive manner to provoke distinct aspects of sickness. It must not be neglected, however, that the interaction might have also been influenced by differences in the purity, potency and elimination of the compounds under study.

### 4.1 Effects of NOD1 and NOD2 agonism

FK565 (0.001–0.003 mg/kg) and MDP (1–3 mg/kg), administered alone, were largely inactive in eliciting sickness. Thus, they failed to significantly decrease locomotion and exploration in the LabMaster system. This finding is in overall agreement with reports that NOD2 activation leads only to a slight decline of locomotion (Fosset et al., 2003; Engeland et al., 2003). Food intake in the LabMaster system remained likewise unaltered by MDP. MDP has been reported to reduce food intake at 1.6 mg/kg in rats, while a dose of 0.6 mg/kg was ineffective (Biberstine and Rosenthal, 1994; Fosset et al., 2003; Langhans et al., 1990). Thus, the dose of 1 mg/kg MDP used here might have been too low to affect ingestion. In addition, murine macrophages are less susceptible to MDP than rat macrophages, indicating species differences in the sensitivity to MDP (Nagao et al., 1990). However, this argument is relativized by the finding that a higher dose of MDP (3 mg/kg) given to double-housed mice outside the LabMaster system failed to cause weight loss within 1 day after treatment. This observation is in keeping with studies in rats in which MDP failed to reduce body weight (Cloutier et al., 2012; Engeland et al., 2003) although weight gain may be decreased (Biberstine and Rosenthal, 1994). FK565 (0.001 mg/kg) reduced food intake by trend when given to single-housed mice, whereas no appreciable weight loss was observed 21 h after injection of a higher dose of FK565 (0.003 mg/kg) in double-housed animals. It has previously been reported that body weight decreases after an injection of 6 mg/kg FK565 (Izumi et al., 1983).

The lack of a sickness response to FK565 and MDP alone was paralleled by a failure of these NOD agonists to significantly augment circulating cytokine levels 3 h after injection. FK565, however, but not MDP, significantly increased circulating corticosterone, which indicates that the NOD1 agonist stimulated the HPA axis, a component of the sickness response (Lenczowski et al., 1997). Since brain cytokine expression was comparable between FK565 and MDP, it appears unlikely that the FK565-evoked rise of plasma corticosterone was mediated by cytokines. Since nitric oxide (NO) participates in the activation of the HPA axis (Bugajski et al., 2004) and FK565 is more potent in inducing NO than MDP (Cartwright et al., 2007), NO may be a mediator of the cytokine-independent HPA axis stimulation due to NOD1 agonism.

As MDP and FK565 were also unable to change body temperature, anxiety-like behavior and SP, we conclude that stimulation of NOD1 and NOD2 alone, with doses of FK565 and MDP that enhance the effects of LPS, is insufficient to evoke an overt sickness response.

### 4.2 Effects of combined NOD1 or NOD2 plus TLR4 agonism in causing sickness

Interaction and crosstalk between the signaling pathways of TLRs and NLRs lead to increased or decreased production of proinflammatory cytokines, depending on the cell type tested (Elinav et al., 2011). Pretreatment of monocytic cells with NOD agonists can facilitate the LPS-induced production of various cytokines (Chamaillard et al., 2003; Fritz et al., 2005; Park et al., 2007; Uehara et al., 2005), and a similar synergistic increase of cytokine production following exposure to NLR and TLR agonists is seen *in vivo* (Parant et al., 1995; Shikama et al., 2011). Furthermore, priming with MDP enhances anaphylactoid reactions and lethality evoked by LPS (Takada and Galanos, 1987; Takada et al., 1990), while intravenous administration of FK565 alone has been reported to elicit signs of septic shock in rats (Cartwright et al., 2007). Priming with MDP can also aggravate the reduction of ingestion and locomotion induced by LPS in rats (Engeland et al., 2003; Langhans et al., 1990), whereas the behavioral effects of combined NOD1 and TLR4 agonism remained unexplored.

The ability of NLR agonism to aggravate and prolong the sickness response to LPS is particularly highlighted by the LabMaster data. Specifically, the low dose of 0.1 mg/kg LPS was able to decrease only locomotion and ingestion, while the combination of FK565 + LPS and MDP + LPS aggravated and prolonged the effects of LPS on all parameters tested (locomotion, exploration, ingestion, SP) and led to a significant decrease of locomotion, exploration (rearing) and food intake for 2–3 days. In contrast, SP was decreased for a shorter period of time.

The LabMaster results also shed some light on the effect of single housing in immune–brain interactions. Housing conditions can modify affective behavior (Painsipp et al., 2011), and single housing made the animals more vulnerable by the PRR agonists. While, in line with the literature (Frenois et al., 2007), novelty-induced locomotion in the OF was not altered 1 day after treatment with 0.1 mg/kg LPS, home cage activity in the LabMaster was decreased for a longer period. Since avoidance of physical activity is a sensitive indicator of illness (Skinner et al., 2009), fatigue and lethargy appear to persist for a longer period than revealed by the novelty-induced locomotion paradigm, although the different testing conditions may also contribute to the different outcomes. The timing of the experiments with regard to the light–dark cycle might be a further explanation for the differences observed inside and outside the LabMaster system. Thus home cage behavior in the LabMaster system was monitored during the whole light–dark cycle, while the behavioral tests were conducted during the light phase when activity levels are generally lower. The SP test has been used to measure anhedonia as an indication of depression-like behavior at a time point when food intake had already normalized (Frenois et al., 2007). However, the LabMaster data indicate that the anorexic effect of LPS outlasts its anhedonic effect. Our observation is backed by other studies in which the duration of LPS-induced sickness has been found to overlap with that of depression-related behavior (Biesmans et al., 2013).

A decrease in locomotion and exploration can also reflect visceral hyperalgesia due to inflammatory processes (Schwartz et al., 2013). In line with this contention, LPS has been shown to evoke acute pain (Kamei et al., 2004) although it has also been reported to induce analgesia via activation of opioid receptors (Yirmiya et al., 1994). Likewise, the cytokine-independent analgesic effect of MDP and FK565 is blocked by the opioid receptor antagonist naloxone (Sato et al., 2010). It thus seems unlikely that the behavioral response to combined NLR and TLR4 agonism reflects hyperalgesia, but this issue needs further investigation.

Enhanced depression-like behavior has been observed in mice 24 h after injection of LPS (0.83 mg/kg) when the sickness behavior has largely vanished (Frenois et al., 2007). As shown here, the dose of 0.1 mg/kg LPS was too low to induce depression-like behavior in the FST, which is in accordance with the literature (Deak et al., 2005). The combination of FK565 or MDP plus 0.1 mg LPS nominally prolonged the time spent immobile in the FST, which attests to a facilitatory effect in the development of depression at this low dose of LPS.

Striking differences between the effects of single and combined administration of NLR and TLR agonists were seen with regard to body temperature. While LPS alone did not induce any change as measured 4 h post-treatment, the combination of FK565 or MDP with 0.83 mg/kg LPS induced overt hypothermia, while the combination with 0.1 mg/kg LPS caused only a slight decrease of body temperature. The thermoregulatory response to LPS in rodents depends on its dose, the route of administration and ambient temperature (Rudaya et al., 2005). At ambient temperatures below thermoneutrality mice develop mild hypothermia at intermediary doses of LPS and excessive hypothermia at high doses, indicative of a septic shock-like condition (Krakauer et al., 2010). The interaction of NLR and TLR agonists in augmenting hypothermia is in line with the aggravation and prolongation of sickness observed in the LabMaster and OF. Thus, the combination of FK565 or MDP plus 0.83 mg/kg LPS decreased novelty-related locomotion (total distance traveled) in the OF 21 h post-treatment compared to LPS. An anxiogenic effect of LPS was uncovered when a lower dose of LPS (0.1 mg/kg), being devoid of an effect on locomotion, was tested. This increase in anxiety-like behavior was more pronounced after treatment with FK565 + LPS, but not MDP + LPS. The seemingly paradox observation that LPS alone increased anxiety-like behavior at the 0.1, but not 0.83 mg/kg dose may be explained by the surmise that any change in anxiety-like behavior is masked by the decrease in locomotion evoked by 0.83 mg/kg LPS.

### 4.3 Potential mechanisms of the synergistic effects of NLR and TLR agonists on sickness

In order to shed light on potential mechanisms whereby FK565 and MDP aggravate LPS-induced sickness, several peripheral and cerebral factors were analyzed. Expression of c-Fos in brain regions known to respond to immune stimulation (Frenois et al., 2007) was used to examine whether the exaggerated sickness response to MDP + LPS (0.83 mg/kg) is reflected by pertinent changes of neuronal activity in the brain. Being the product of an immediate early gene, c-Fos is rapidly but transiently expressed following neuronal activation (Rivest and Laflamme, 1995) and therefore was measured 3 h after immune stimulation. LPS alone induced c-Fos expression in brainstem-derived ascending pathways to forebrain immune-responsive nuclei (Gaykema and Goehler, 2011). Priming with MDP enhanced the number of c-Fos positive neurons in an additive or synergistic manner depending on the area examined. Thus the increase of c-Fos expression in the BNSTd and CeA was additive, while synergistic increases of c-Fos expression were observed in the BNSTv, PVN, insula and SO. These observations indicate that the synergistic effect of NOD2 and TLR4 stimulation on the sickness response is related to enhanced neuronal activation in relevant brain nuclei. Of particular interest was the observation that LPS, administered in the absence or presence of MDP, decreased the number of c-Fos positive cells in the dentate gyrus of the hippocampus, which has been associated with a decrease of exploratory behavior following treatment with LPS (Gaykema and Goehler, 2011).

Although the HPA axis participates in the sickness response (Lenczowski et al., 1997), the current results indicate that the HPA axis did not contribute to the aggravation of sickness by combined NLR + TLR agonism, since neither FK565 nor MDP augmented the LPS-induced rise of plasma corticosterone, both in the absence of stress and following exposure to tail suspension stress.

In contrast, proinflammatory cytokines in the plasma and brain are very likely to mediate the exacerbation of sickness due to NLR plus TLR agonism. Consistent with the interaction of FK565 and MDP with LPS in innate immune cells (Le Contel et al., 1993; Netea et al., 2005; Wang et al., 2001; Wolfert et al., 2002), the LPS-evoked rise of plasma cytokines 3 h post-treatment was synergistically enhanced by priming with FK565 or MDP.

In parallel with their peripheral induction, the cerebral expression of IFN-γ and IL-6, was synergistically enhanced by FK565 + LPS and MDP + LPS, while the increase of cerebral IL-1β and TNF-α mRNA expression was rather additive. Thus, the effects of NOD agonists to prime the production of proinflammatory cytokines in response to LPS exhibit different patterns of interaction in the periphery and brain, depending on the compounds investigated. While this interaction is of a primarily synergistic nature in the periphery, the interaction in the brain can either be of an additive or synergistic manner. This different pattern of interaction is also reflected by different time courses of cytokine induction in the periphery and brain. While the PRR-evoked increase in plasma cytokines had largely waned 1 day post-treatment, the cerebral expression of IL-1β and TNF-α mRNA was still elevated. The most striking difference was seen with IL-6, the plasma levels of which remained elevated 1 day after treatment with LPS, MDP + LPS and FK565 + LPS, whereas the cerebral expression of IL-6 mRNA was reduced by these treatments, as previously described for LPS (Andre et al., 2008; Bay-Richter et al., 2011).

Collectively, our findings suggest that the sickness response to combined NLR and TLR agonism is initiated by immune stimulation which in turn activates secondary mechanisms that drive illness. Kynurenine may play such a role, given that its plasma level was significantly more enhanced by the combination treatments than by LPS alone and remained significantly elevated 1 day post-treatment. In line with this contention, blockade of IDO decreases kynurenine levels and abrogates LPS-induced depression-like behavior without changing brain cytokine expression (O’connor et al., 2009). Proinflammatory cytokines, particularly IFN-γ and TNF-α, activate IDO and lead to the conversion of tryptophan to kynurenine which in rodents elicits depression-like behavior (O’connor et al., 2009). The increase in plasma tryptophan seen here contrasts with a decrease of tryptophan seen in other studies (O’connor et al., 2009) but may be related to the TST employed 30 min before blood sampling, given that stress can increase peripheral tryptophan levels in rodents, but the underlying mechanisms are not understood (Dunn, 1988; Malyszko et al., 1995). It is, however, emerging that not tryptophan depletion but kynurenine production contributes to the behavioral effects of immune activation (O’connor et al., 2009). Although peripheral tryptophan may decrease in response to LPS, the availability of tryptophan to the brain remains unchanged and brain tryptophan may even increase (O’connor et al., 2009).

Both NOD1 and NOD2 agonists enhanced the sickness response to LPS in a grossly similar manner, which is surprising in view of the differences in the expression, distribution and function of these PRRs and in the sources and affinities of their agonists (Dharancy et al., 2010; Hsu et al., 2008; Masumoto et al., 2006; Sabbah et al., 2009). Thus, NOD1 is more widely expressed than NOD2 and occurs in peripheral and cerebral tissues (Inohara et al., 1999), while NOD2 expression is largely restricted to monocytes (Ogura et al., 2001). In line with these findings, cytokine levels were generally higher after treatment with MDP + LPS, together with the highest corticosterone levels and the kynurenine/tryptophan ratio in this group 1 day post-treatment, pointing to a relationship between cytokine expression, corticosterone release and kynurenine formation. In contrast, the behavioral effects were tendentially more pronounced after treatment with FK565 ± LPS. These disparities may arise from differential interactions of NOD1 and NOD2 with TLR4 at the blood–brain interface. On the one hand, NOD1, but not NOD2, is expressed in the choroid plexus and other circumventricular organs (Inohara et al., 1999), which may also account for the particular ability of FK565 to enhance circulating corticosterone. On the other hand, the cerebral effects of peripherally injected LPS may be mediated by TLR4 on CNS-resident cells and be independent of systemic cytokine effects (Chakravarty and Herkenham, 2005; Murray et al., 2011). In addition, LPS is able to induce a transient rise of intracellular calcium in microglial cells of the area postrema, while MDP is devoid of such an effect, again pointing to absent NOD2 expression at this circumventricular organ (Wuchert et al., 2008).

### 4.4 Conclusions

The current study shows that NOD1 and NOD2 activation alone has only minor effects on cytokine production and sickness behavior but potently synergizes with TLR4 stimulation in aggravating and prolonging illness. Analysis of the potential mechanisms led us to conclude that the aggravation of sickness is associated with enhanced production of proinflammatory cytokines in the periphery and brain, increased kynurenine formation and activation of immune responsive brain nuclei. Further studies are warranted to analyze whether NOD1 and TLR4 interact with each other primarily at the blood–brain interface while NOD2 and TLR4 synergism occurs primarily in hematopoietic cells. Under conditions of infection or an imbalance in microbiota-host interaction, NLRs and TLRs are likely to be targeted in parallel by an expanded number of PRR agonists. It remains to be investigated whether the concentrations of endogenous PRR agonists occurring in infection give rise to a similar synergism of NLRs and TLRs as seen here with FK565, MDP and LPS. We propose that the interaction of NLRs and TLRs in boosting a multidimensional sickness response reflects an important immunological and neurobiological mechanism of protection from microbial invasion.

## Figures and Tables

**Table 1:** Effects of LPS (0.1 mg/kg), MDP (3 mg/kg) + LPS and FK565 (0.003 mg/kg) + LPS on depression-like behavior in the FST and TST 1 day post-treatment in male mice.

	VEH	LPS	MDP + LPS	FK565 + LPS
*FST*				
Immobility	237.70 ± 30.34	242.14 ± 22.55	285.91 ± 13.11	292.27 ± 9.16
Swimming	108.25 ± 28.95	102.15 ± 21.77	65.71 ± 13.06	53.95 ± 9.57
Climbing	14.07 ± 2.04	15.75 ± 4.38	8.40 ± 2.15	13.81 ± 4.86

*TST*				
Immobility	261.25 ± 11.71	241.93 ± 7.33	252.43 ± 12.72	232.01 ± 10.19
Swinging	40.58 ± 6.89	55.13 ± 7.48	60.19 ± 15.74	56.94 ± 6.41
Curling	58.19 ± 5.49	62.96 ± 5.79	47.40 ± 6.55	71.07 ± 8.36

Mice were injected i.p. with saline (VEH), LPS, MDP + LPS or FK565 + LPS in two separate experiments. The mice subjected to the FST (21 h post-treatment) were singly housed, while the mice exposed to the TST (25.5 h post-treatment) were kept in groups of 2. The duration of immobility, swimming, climbing, swinging and curling during the 6 min test session is expressed in seconds. The values are means ± SEM, *n* = 7–8.
